# Thoracic pain in patients with chronic interstitial lung disease—an underestimated symptom

**DOI:** 10.3389/fmed.2023.1147555

**Published:** 2023-05-05

**Authors:** Manuela J. Scherer, Sandra Kampe, Jonas Fredebeul-Beverungen, Gerhard Weinreich, Ulrich Costabel, Francesco Bonella

**Affiliations:** ^1^Department of Anaesthesiology and Pain Medicine, University Medicine Essen—Ruhrlandklinik, University Duisburg-Essen, Essen, Germany; ^2^Department of Anaesthesiology and Intensive Care Medicine, University Hospital Magdeburg, Otto von Guericke University Magdeburg, Magdeburg, Germany; ^3^Pneumology Department, University Medicine Essen—Ruhrlandklinik, University Duisburg-Essen, Essen, Germany; ^4^Pneumology Department, Center for Interstitial and Rare Lung Diseases, University Medicine Essen—Ruhrlandklinik, University Duisburg-Essen, Essen, Germany

**Keywords:** chronic interstitial lung disease, thoracic pain, prevalence of thoracic pain, risk factors, quantitative sensory testing

## Abstract

**Introduction:**

Prevalence and predisposing factors for the development of thoracic pain (TP) in patients with chronic interstitial lung disease (cILD) are largely unknown. Underestimation and insufficient therapy of pain can lead to worsened ventilatory function. Quantitative sensory testing is an established tool for characterization of chronic pain and its neuropathic components. We investigated frequency and intensity of TP in cILD patients and the potential association with lung function and quality of life.

**Materials and methods:**

We prospectively investigated patients with chronic interstitial lung disease to analyze risk factors for the development of thoracic pain and quantify thoracic pain through quantitative sensory testing. In addition, we studied the relationship between pain sensitivity and lung function impairment.

**Results:**

Seventy-eight patients with chronic interstitial lung disease and 36 healthy controls (HCs) were included. Thoracic pain occurred in 38 of 78 patients (49%), most frequently in 13 of 18 (72%, *p* = 0.02) patients with pulmonary sarcoidosis. The occurrence was mostly spontaneous and not related to thoracic surgical interventions (76%, *p* = 0.48). Patients with thoracic pain showed a significant impairment of mental well-being (*p* = 0.004). A higher sensitivity to pinprick stimulation during QST can be observed in patients with thoracic pain (*p* < 0.001). Steroid treatment was associated with lower sensitivity within thermal (*p* = 0.034 and *p* = 0.032) and pressure pain testing (*p* = 0.046). We observed a significant correlation between total lung capacity and thermal (*p* = 0.019 and *p* = 0.03) or pressure pain sensitivity (*p* = 0.006 and *p* = 0.024).

**Conclusion:**

This study was performed to investigate prevalence, risk factors and thoracic pain in patients with chronic interstitial lung disease. Thoracic pain mostly occurs spontaneous as a frequent symptom, and seems to be an underestimated symptom in patients with chronic interstitial lung disease, especially those with pulmonary sarcoidosis. Timely identification of thoracic pain may allow starting symptomatic treatment at early stage, before impairment in quality of life occurs.

**Clinical Trial Registration:**

https://www.drks.de/drks_web/, Deutsches Register Klinischer Studien (DRKS) DRKS00022978.

## 1. Introduction

Interstitial lung diseases (ILDs) are chronic diseases characterized by diffuse inflammation and/or fibrosis of the lung parenchyma, leading to restrictive ventilatory impairment, progressive dyspnea and respiratory insufficiency (1–3). The diagnosis is mainly based on clinical and high resolution computed tomography (HRCT) findings (1). Quality of life in IPF/ILD patients is impaired, especially due to cough and shortness of breath (4–7).

Although the exact frequency is unknown, a subgroup of IPF/ILD patients develops thoracic pain (TP), sometimes migrating, sometimes localized, which is difficult to classify especially in absence of a history of thoracotomy or any other thoracic intervention, and is often not associated with cough (8). Inadequate recognition and management of TP could lead to inspiration limitation, contraction of expiratory muscles, and consecutively to enhanced restriction, aggravating hypoxemia in advanced stage of disease (9).

Quantitative sensory testing (QST), developed within the framework of the German Research Network “Pain” and used worldwide since 2002 as a routine tool to assess pain (10), is a validated tool to assess sensory and nociceptive perception, as well as identifying neuropathic components of pain (11, 12). Patient responses to different physiological stimuli are recorded to quantify and qualify somatosensory integrity and pain sensitivity (13).

Aim of this study was to systematically investigate frequency and intensity of thoracic pain in cILD patients and explore possible correlations with lung function impairment as well as deterioration of quality of life.

## 2. Materials and methods

### 2.1. Study subjects

The study prospectively investigated patients with cILD and a control group of healthy controls (HCs). Study participants were consecutively recruited among patients with cILD followed at the Ruhrlandklinik between April 2017 and November 2019.

Inclusion criteria were an interstitial lung disease diagnosed according to American Thoracic Society (ATS)/European Respiratory Society (ERS) criteria 2013 (14). Patients with unclassifiable ILD, incomplete data set or acute thoracic pain caused by any recent events (embolism, pneumothorax) or surgical interventions (including open lung biopsy) during the past 6 month, were excluded. Exclusion of acute causes was based on Chest X ray and complete lung function test during routine follow up visits.

As a control group, we investigated HCs with age > 18 years, no pre-existing lung diseases, no chronical pain syndromes, no pre-existing analgesic medication or neurological conditions such as polyneuropathy. Healthy controls were recruited among the employees of our institution after an accurate anamnestic screening for underlying diseases or conditions associated with chronic thoracic pain. The study was approved by the local ethics committee of the Medical Faculty of the University Duisburg-Essen (16-7028-BO), and registered in the German register of clinical studies (DRKS00022978). Written informed consent was obtained from all participants.

### 2.2. Questionnaires

All questionnaires were collected prior to performing QST. The Short Form 12 (SF-12) questionnaire was employed for assessing health-related QoL regarding physical and mental well-being (15). The painDETECT questionnaire was used to evaluate potential neuropathic pain (16). The painDETECT total score ranges between 0 and 38 and denotes the possibility of a neuropathic pain component being present (<13 very unlikely, 13–18 likely, >19 certainly). The number of pain areas was recorded by the body scheme of the painDETECT questionnaire.

### 2.3. Thoracic pain definition and quantitative sensory testing

TP was defined as persistent or intermittent pain ≥1 in the numeric rating scale (NRS), the most frequently used pain assessment scale (17).

QST is an established psychophysical test protocol for the quantitative evaluation of somatosensory function (18). The test is based on standardized somatosensory stimuli for which participant responses are recorded. Thirteen parameters can be obtained from seven separate test procedures involving nociceptive and non-nociceptive sensations (10). The same calibrated thermal and mechanical stimuli are always set in the same test sequence. For the present study, the following 10 QST-parameters were obtained in the given order: cold detection threshold (CDT), warm detection threshold (WDT), cold pain threshold (CPT), heat pain threshold (HPT), mechanical detection threshold (MDT), mechanical pain threshold (MPT), mechanical pain sensitivity (MPS), dynamic mechanical allodynia (DMA), wind-up ratio (WUR), and pressure pain threshold (PPT) (Extensive description in Supporting information). The correlation of the scores with impairment of sensitivity and pain perception can vary, for some scores being positive (WDT, HPT, MDT, MPS, DMA, and WUR) and for others negative (CDT, CPT, MPT, and PPT).

QST was performed by two trained examiners. Participants were trained in QST procedures by performing all tests on one hand, once. Subsequently, QST measurements were performed on both sides of the thorax. Patients were instructed not to look at the test area.

#### 2.3.1. Thermal measurement

Thermal measurement was performed with a Medoc TSA 2001-II device (Medoc, Israel) (19, 20). The contact area of the thermode was 30 × 30 mm. Baseline temperature was 32°C for skin adaption. The subject was requested to stop the stimulus with linearly increasing intensity (1°C/s) immediately when perceiving the onset of cooling (CDT), warming (WDT), or the additional sensation of burning, stinging, drilling or pulling (CPT, HPT). The measurement was terminated by the patient through pressing a button, or when reaching the cut-off temperatures of 0°C and 50°C. Each temperature threshold was obtained three times per target area. Thresholds were calculated as the arithmetic mean temperature of the three consecutive measurements (18).

#### 2.3.2. Mechanical detection threshold

MDT was measured with modified von Frey filaments made of optic glass fibers (OptiHair2-Set, Marstock Nervtest, Germany) that exert forces between 0.25 and 512 mN increasing by a factor of two from filament to filament (21, 22). The contact area of the filaments was a small epoxy beat with a diameter of 0.30–0.45 mm. Participants were asked to close their eyes, so that they could not observe the application of filaments, and were instructed to immediately report any perceived touch sensation within the target area. The force of the filaments was incrementally increased until the participant reported the first touch sensation. The force of the last filament used was noted as the first suprathreshold value. After this, filaments were applied in incrementally decreasing force until the patient did not report a touch perception. The force of the last filament was noted as the first infrathreshold value (23). This procedure was repeated five times per target area. The threshold was determined as the geometric mean of five supra-and infrathreshold values (18).

#### 2.3.3. Mechanical pain threshold

MPT was measured with a PinPrick-Set (MRC Systems GmbH, Germany) that exert forces between 8 and 512 mN increasing by a factor of two from pinprick to pinprick (24). The contact area of the pinpricks is 0.2 mm diameter. The sensation of pinpricks is produced by the weight of the needle resting on the skin of patient. Patients were instructed to report whether the touch of a pinprick evoked the sensation sharpness, or not. In increasing forces, the force of the first pinprick described as sharp had to be noted as the first suprathreshold value, followed by descending stimuli until the first pinprick is only a touch, noted as infrathreshold value. As for the MDT, five ascending and descending series of stimuli were performed per target area. The MPT was calculated as the geometric mean of five infra-and suprathreshold values (18).

#### 2.3.4. Mechanical pain sensitivity/dynamic mechanical allodynia

Using needle stimulators of different intensities, a stimulus–response curve of MPS was generated (23). Seven different stimulus intensities were applied in a randomized sequence including each stimulus intensity five times per area. The patient evaluated the individual pain intensities directly after each individual stimulus according to a numeric rating scale between 0 and 100. DMA was examined according to the same test scheme as described for MPS. A moving touch stimulus (cotton swab, Q-tip, brush), which normally does not lead to painful perception, was applied between the needle stimuli. Each of these three non-noxic stimuli was applied five times per area. A total of 50 stimuli (touch and needle stimulus) were applied on both sides of the thorax and the painfulness was recorded numerically. As a measure for the sensitivity to pain, the geometric mean value of pain ratings for needle (MPS) and touch (DMA) stimuli was calculated (18).

#### 2.3.5. Wind-up ratio

WUR was determined with a pinprick of 128 mN. A single stimulus alternated with a train of 10 pinprick stimuli (1/s) within an area of 1 cm^2^. The single stimulus and the stimulus train were rated by the patient on a numeric rating scale between 0 and 100, separately. The procedure was repeated five times. The wind-up ratio was calculated as the arithmetic mean pain rating of the five trains divided by the arithmetic mean pain rating of the five single stimuli (18).

#### 2.3.6. Pressure pain threshold

PPT was measured using a pressure gauge device (FDN 200, Wagner Instruments, United States) with a contact area of 1 cm^2^ and pressure limit of 20 kg/cm^2^, equivalent to 2,000 kPa. The algometer was applied to the thenar of the respective test side, as testing on the chest is not possible due to the insufficiently large contact area to the muscles in the intercostal space. The application was made manually, with an increasing force of 50 kPa/s, corresponding to 0.5 kg/cm^2^/s. Participants were asked to indicate the onset of a burning, stinging, drilling or pulling sensation. Application of pressure was stopped on feedback and the force reached was recorded as the threshold (25, 26). The procedure was repeated three times per target area. Pressure pain threshold was calculated as the arithmetic mean of these three measurements (18).

### 2.4. Pulmonary function tests and blood gas analysis

Measurements including FVC, forced expiratory volume in 1 s (FEV1), TLC and DLCO were carried out with Vyntus^®^ SPIRO or Vyntus^®^ PNEUMO and Masterscreen Body from Carefusion/Vyaire Medical. Blood gas analysis was performed with ABL from Radiometer to measure arterial oxygen tension, arterial carbon dioxide tension, arterial oxygen saturation, and alveolar-arterial oxygen tension difference. Lung function tests were performed at the time of QST.

### 2.5. Statistics

Variables distribution was calculated by using Kolmogorov–Smirnov test. Descriptive statistics (frequency and mean ± standard deviation) were performed. Non normally distributed variables are presented as median with interquartile range (IQR). Sample size was calculated based on a number of 360 patients with a new diagnosis of ILD per year at our institution and the fact that up to 10% of them are expected to have TP not dependent on surgical procedures (estimated population size 36). The minimum sample size of ILD patients with TP is 33 with a confidence level of 95% (95%CI) and a margin of error of 5%.

Comparison between cILD patients and HCs were tested using the Mann–Whitney *U*-test or Student’s *t*-test for continuous variables and chi-square test for categorical variables. Correlations between continuous variables were calculated by using Pearson or Spearman correlation tests. We considered *p* ≤ 0.05 to be statistically significant. Statistical analysis was performed with SPSS 27.0 (SPSS Inc., Chicago, United States).

## 3. Results

### 3.1. Studied subjects

We enrolled 81 patients with cILD followed at our Institution between April 2017 and November 2019, mostly in the outpatient clinic. As a control group, 36 healthy subjects were included. Three patients were excluded from testing because QST or lung function were not completed. cILD patients and HCs differed significantly in female percentage, age and pack/years (Table 1).

**Table 1:** Demographic data of patients with chronic interstitial lung disease and healthy controls.

Group	Patients with chronic ILD (*N* = 78)	Healthy controls (*N* = 36)	*p*-value
Women	25 (32%)	20 (56%)	0.017*
Age (year), mean ± SD	65.0 ± 13.0	45.8 ± 14.9	<0.001
Smoking years (pack years)	8 (0.0–30)	0.0 (0.0–7)	0.002
Alcohol units per week	0.0 (0.0–3)	2 (0.0–5)	0.001
Respiratory comorbidities			
COPD	5 (6%)	–^a^	–
Asthma	7 (9%)	–^a^	–
Pre-existing treatments with analgesic or potential analgesic effect	36 (46%)	–^b^	–
Non-opioid analgesics	24 (31%)	–^b^	–
Opioids	12 (15%)	–^b^	–

Otherwise indicated, values are expressed as median and IQR. Mann–Whitney-*U*-test for comparison was used.

* *T*-test for comparisons was used.

a Pre-existing pulmonary disease was an exclusion criterion for healthy controls.

b The intake of analgesic medication was an exclusion criterion for healthy controls.

### 3.2. Frequency of thoracic pain and correlation with demographics and clinical characteristics

TP occurred in 38 (48.7%) of 78 examined patients with cILD (Table 2). Time since initial diagnosis of cILD did not differ between patients with and without thoracic pain (*p* = 0.07; Table 2). TP occurred more frequently in patients with pulmonary sarcoidosis (72%) (*p* = 0.02) than in those with other cILDs (47%), and less frequently in patients with EAA (29%) (*p* = 0.07). TP was spontaneous in 76% of cases, related to previous thorax interventions in 5%, and of unknown origin (not indicated by the patients) in 19% of cases.

**Table 2:** Demographic data of patients with chronic interstitial lung disease with and without thoracic pain.

Group	ILD patients	ILD patients	*p*-value
	With thoracic pain	Without thoracic pain	
Patients	38 (49%)	40 (51%)	
Women	15 (39%)	10 (25%)	0.17
Age (year)	64.0 ± 12.8	65.9 ± 13.3	0.37*
Smoking years (pack years) (median, IQR)	2 (0.0–31)	13 (0.0–30)	0.47*
Alcohol units per week (median, IQR)	0.0 (0.0–1)	0.0 (0.0–5)	0.47*
ILD disease duration prior to QST (month)	30 ± 70	28 ± 42	0.07
Diagnosis			
IPF	11 (29%)	12 (30%)	0.92
NSIP	4 (11%)	2 (5%)	0.36
EAA	5 (13%)	12 (30%)	0.07
DIP	2 (5%)	1 (3%)	0.53
Sarcoidosis^a^	13 (34%)	5 (13%)	0.02
Other ILD	3 (8%)	8 (20%)	0.13
Respiratory comorbidities			
COPD	3 (8%)	2 (5%)	0.6
Asthma	4 (11%)	3 (8%)	0.64
Lung function testing			
Oxygen dependency (L/min)	2.7 ± 1.0	3.5 ± 1.4	0.34
TLC (L)	5.2 ± 1.3	5.0 ± 1.5	0.49
TLC (%)	82.1 ± 16.7	74.8 ± 16.9	0.06
FEV1 (%)	69.9 ± 19.6	73.1 ± 18.4	0.6
IVC (L)	2.9 ± 1.2	2.9 ± 1.1	1
IVC (%)	75.5 ± 17.9	73.8 ± 20.6	0.62
Tiffeneau index (%)	98.9 ± 14.5	107.6 ± 13.2	0.015
DLCO (%)	49.3 ± 17.5	41.5 ± 17.7	0.11
paO_2_	73.1 ± 14.8	70.8 ± 10.9	0.12
Previous interventions			
No intervention	15 (39%)	19 (48%)	0.48
Transbronchial biopsy	15 (39%)	17 (43%)	0.79
Surgical lung biopsy	8 (21%)	4 (10%)	0.18
SF-12			
Physical health summary scale (median, IQR)	31 (24–39)	34 (26–48)	0.11*
Mental health summary scale (median, IQR)	47 (34–57)	56 (50–59)	0.004*
painDETECT (median, IQR)	7 (3–12)	0.0 (0.0–4)	<0.001*
Areas of pain (median, IQR)	2 (1–4)	1 (0.0–3)	<0.001*
Pre-existing treatments with analgesic or potential analgesic effect			
Non-opioid analgesics	17 (45%)	7 (18%)^b^	0.009
Opioids	7 (18%)	5 (13%)^b^	0.47
Steroids	15 (39%)	17 (43%)	0.79
Tricyclics	2 (0.05%)	1 (0.03%)	0.59
Onset of pain symptoms			
Spontaneously	29 (76%)		
After intervention	2 (5%)		
Statement not possible	7 (18%)		

Otherwise indicated, values are expressed as mean ± SD. *T*-test for comparisons was used.

IPF, idiopathic pulmonary fibrosis; NSIP, nonspecific interstitial pneumonia; EAA, exogenous allergic alveolitis; DIP, desquamative interstitial pneumonia; ILD, interstitial lung disease; COPD, chronic obstructive pulmonary disease; TLC, total lung capacity; FEV_1_, forced expiratory volume in 1 s; IVC, inspiratory vital capacity; DLCO, diffusing capacity for carbon monoxide; paO_2_, oxygen partial pressure.

* Mann–Whitney-*U*-test for comparison was used.

a Of them, 5 patients had stage I, 12 patients had stage II, and 1 had stage III.

b Treatment with analgesic medication in patients without thoracic pain was related to other reasons like back pain or joints (rheumatoid arthritis and arthrosis).

There were no differences according to gender, age, pack/years or alcohol units per week between cILD patients with (TP+) and without thoracic pain (TP−). The intake of non-opioid analgesics was higher in TP+ compared to TP− patients (17 vs. 7, *p* = 0.009), the intake of opioids did not differ between the groups. Furthermore, we did not detect any difference in frequency of previous interventions between TP+ and TP− (Table 2).

### 3.3. Questionnaires

In the physical health summary scale, we did not observe any difference between TP+ and TP− patients, whereas mental health score was significantly more impaired in TP+ compared to TP− patients (median 47, IQR 34–57 vs. 56, IQR 50–59; *p* = 0.004; Table 2). The total score of the pain detect questionnaire was significantly higher in TP+ patients (*p* < 0.001) but did not reach values that are indicative of neuropathic pain. The number of pain areas was significantly higher in TP+ patients (*p* < 0.001) ranging from 0 to 7 pain areas without differences in localization of pain.

### 3.4. Quantitative sensory testing

TP+ patients had higher MPS compared to TP− on both body sides and a significant difference in MPT (*p* = 0.008) and DMA (*p* = 0.012) on the left body side reflecting a higher sensitivity to pinprick stimulation. Measurements of CDT, WDT, CPT, HPT, MDT, WUR, and PPT did not differ between TP+ and TP− patients (Table 3).

**Table 3:** Comparison of quantitative sensory testing in patients with cILD with and without thoracic pain.

Group	ILD patients with thoracic pain	ILD patients without thoracic pain	*p*-value
*N* (%)	38 (49%)	40 (51%)	
Right body side			
CDT (cold detection threshold) [°C]	29 (25–30)	29 (27–30)	0.61
WDT (warm detection threshold) [°C]	37 (35–39)	36 (35–38)	0.31
CPT (cold pain threshold) [°C]	11 (3–24)	2 (0.0–25)	0.31
HPT (heat pain threshold) [°C]	47 (42–50)	48 (44–50)	0.54
MDT (mechanical detection threshold) [mN]	7 (4–17)	5 (2–10)	0.13
MPT (mechanical pain threshold) [mN]	30 (10–62)	34 (20–95)	0.09
MPS (mechanical pain sensitivity)	9 (5–19)	2 (0.7–7)	<0.001
DMA (dynamic mechanical allodynia)	0.0 (0.0–0.4)	0.0 (0.0–0.0)	0.21
WUR (wind-up ratio)	0.3 (0.2–0.5)	0.2 (0.1–0.5)	0.06
PPT (pressure pain threshold) [kg/cm^2^]	6 (5–8)	7 (6–9)	0.25
Left body side			
CDT (cold detection threshold) [°C]	29 (27–30)	30 (27–30)	0.91
WDT (warm detection threshold) [°C]	37 (35–38)	36 (34–38)	0.22
CPT (cold pain threshold) [°C]	19 (5–26)	5 (0.0–26)	0.10
HPT (heat pain threshold) [°C]	45 (41–49)	47 (43–49)	0.58
MDT (mechanical detection threshold) [mN]	7 (3–18)	6 (3–15)	0.66
MPT (mechanical pain threshold) [mN]	23 (11–42)	50 (22–116)	0.008
MPS (mechanical pain sensitivity)	10 (4–18)	2 (0.4–5)	<0.001
DMA (dynamic mechanical allodynia)	0.0 (0.0–0.7)	0.0 (0.0–0.0)	0.012
WUR (wind-up ratio)	0.4 (0.3–0.6)	0.3 (0.2–0.6)	0.38
PPT (pressure pain threshold) [kg/cm^2^]	6 (5–8)	7 (5–8)	0.45

Otherwise indicated, values are expressed as median and IQR. Mann–Whitney-*U*-test for comparisons was used.

Patients under corticosteroid treatment had lower sensitivity within thermal testing and pressure pain testing compared to patients without steroids. Differences in HPT were significant on the right side (median 49, IQR 44–50°C vs. 46, IQR 41–48°C, *p* = 0.034) and left side (median 49, IQR 43–49°C vs. 44, IQR 40–47°C, *p* = 0.032) of the body, while CPT was only significant on the right side (median 0.5, IQR 0–23°C vs. 16, IQR 5–25°C, *p* = 0.032) and PPT only significant on the left side (median 7, IQR 5–9 kg/cm^2^ vs. 7, IQR 5–9 kg/cm^2^, *p* = 0.046).

### 3.5. Correlation with lung function impairment

**Figure 1:**
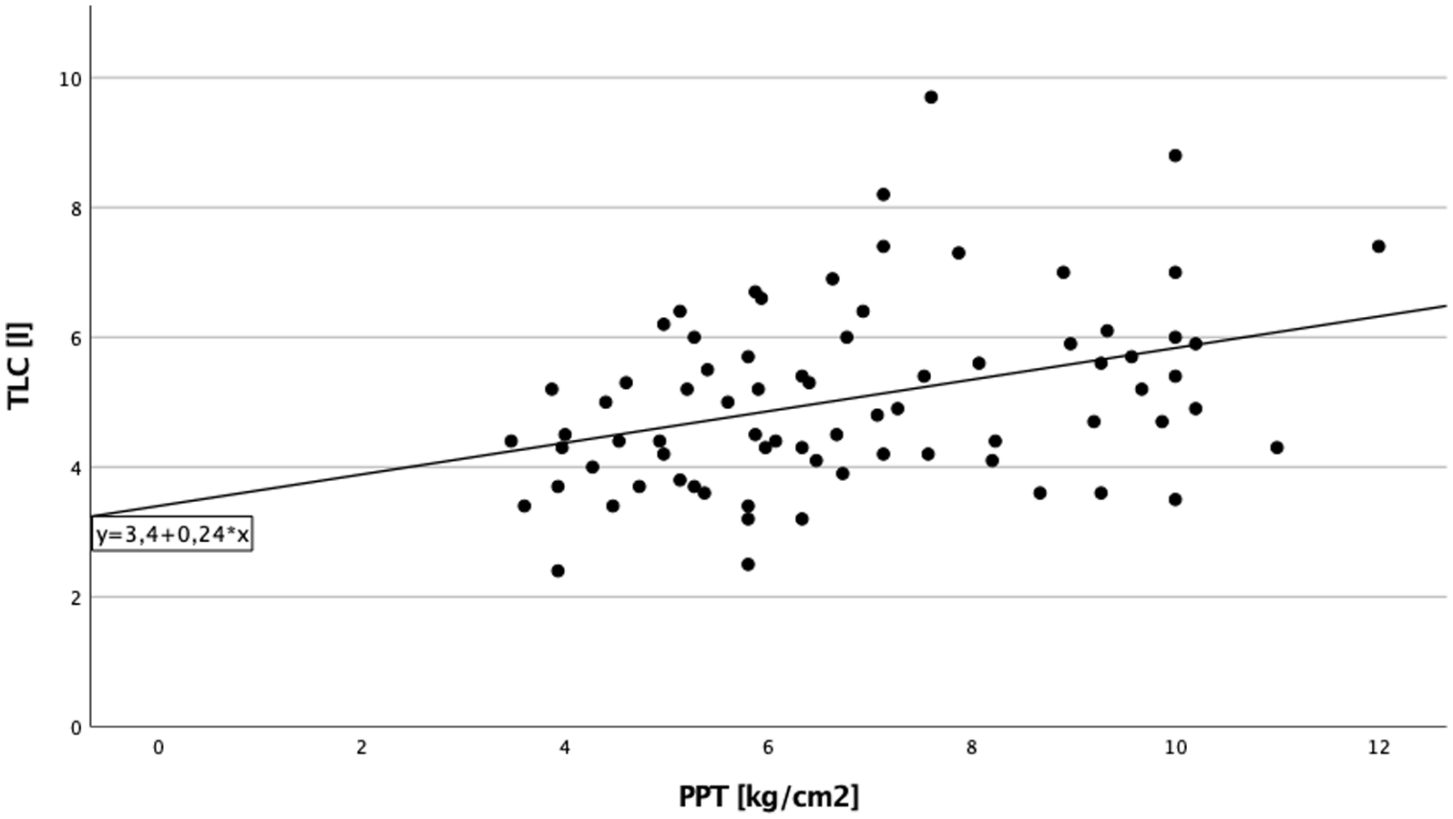
Total lung capacity and pressure pain threshold cILD-patients. Correlation between total lung capacity and pressure pain threshold on the right side of the body in all patients with chronic interstitial lung disease. Higher values in total lung capacity are positively correlated with higher results in pressure pain threshold, and Spearman’s rank correlation coefficient is *r*_S_ = 0.371 (*p* = 0.001), which describe a lower sensitivity to pain.

**Figure 2:**
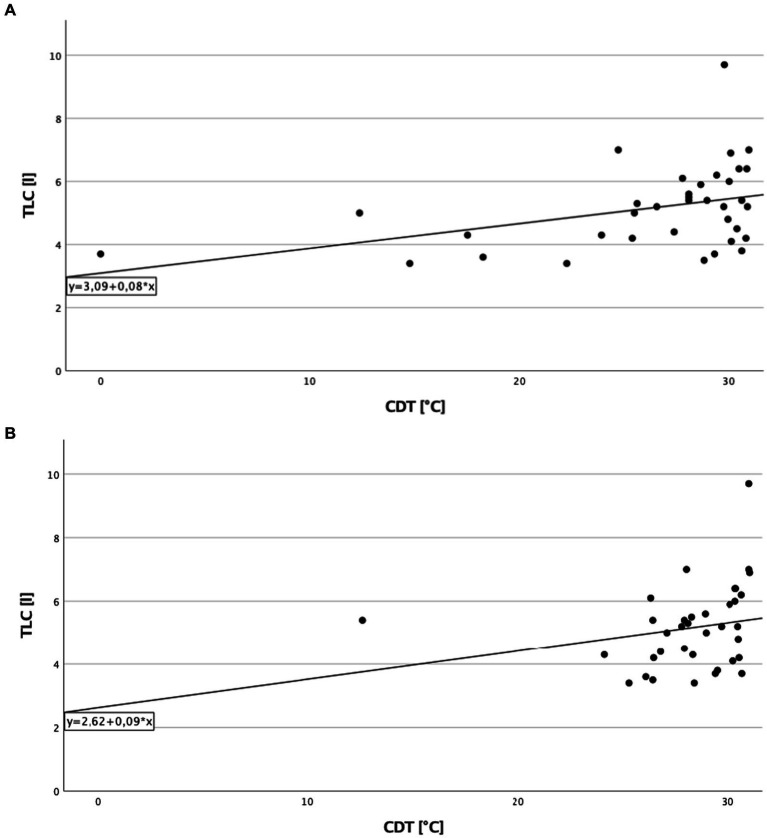
Total lung capacity and cold detection threshold in patients with thoracic pain. **(A)** On the right side of the body. Correlation of total lung capacity and cold detection threshold on the right side in patients with thoracic pain. Higher values in total lung capacity are positively correlated with higher results in cold detection threshold, and Spearman’s rank correlation coefficient is *r*_S_ = 0.388 (*p* = 0.019). **(B)** On the left side of the body. Correlation of total lung capacity and cold detection threshold on the left side in patients with thoracic pain. Higher values in total lung capacity are positively correlated with higher results in cold detection threshold, and Spearman’s rank correlation coefficient is *r*_S_ = 0.362 (*p* = 0.030).

**Figure 3:**
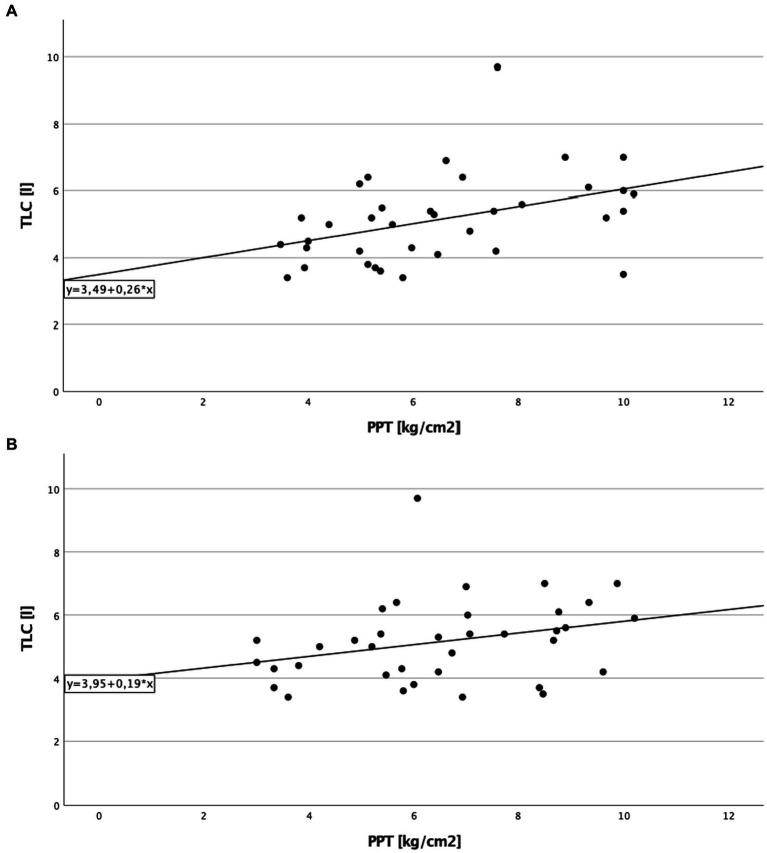
Total lung capacity and pressure pain threshold in patients with thoracic pain. **(A)** On the right side of the body. Correlation of total lung capacity and pressure pain threshold on the right side in patients with thoracic pain. Higher values in total lung capacity are positively correlated with higher results in pressure pain threshold, and Spearman’s rank correlation coefficient is *r*_S_ = 0.452 (*p* = 0.006), which describe a lower sensitivity to pain in association to better lung function testing. **(B)** On the left side of the body. Correlation of total lung capacity and pressure pain threshold on the left side in patients with thoracic pain. Higher values in total lung capacity are positively correlated with higher results in pressure pain threshold, and Spearman’s rank correlation coefficient is *r*_S_ = 0.375 (*p* = 0.024).

A significant direct correlation between total lung capacity (TLC) and PPT on the right side of the body in cILD patients was found (*p* = 0.001; *r*_S_ = 0.371), independent from the presence or absence of TP (Figure 1). In TP+ patients, TLC directly correlated with CDT (*p* = 0.019; *r*_S_ = 0.388 and *p* = 0.030; *r*_S_ = 0.362) and PPT (*p* = 0.006; *r*_S_ = 0.452 and *p* = 0.024; *r*_S_ = 0.375) on both sides of the body, meaning that the higher are the values of TLC, the better was cold sensitivity and the lower was the pressure pain sensitivity (Figures 2, 3).

### 3.6. Comparison to healthy controls

Significant differences between cILD patients with thoracic pain (TP+) and HCs could be observed in CDT [*p* = 0.021 (right); *p* = 0.002 (left)], MPS [*p* = 0.001 (right); *p* = 0.002 (left)], DMA [*p* = 0.006 (right); *p* = 0.003 (left)] and PPT [*p* = 0.007 (right); *p* = 0.047 (left)] on both body sides and in HPT (*p* = 0.045) only on the left body side (Table 4). TP+ patients showed lower sensitivity within thermal testing, increased values on numeric rating scale during pinprick stimulation and higher sensitivity to pressure pain. Between cILD patients without thoracic pain (TP−) and HCs we found significant differences in HPT [*p* = 0.025 (right); *p* = 0.005 (left)] and MPT [*p* = 0.001 (right); *p* < 0.001 (left)] on both sides and in CDT (*p* = 0.005), CPT (*p* = 0.009) and MPS (*p* = 0.017) on the left side of the body (Table 5). TP− patients showed lower sensitivity within thermal testing, delayed sensation of sharpness and decreased values on numeric rating scale during pinprick stimulation compared to HCs.

**Table 4:** Comparison of quantitative sensory testing in patients with cILD with thoracic pain and healthy controls.

Group	ILD patients with thoracic pain	Healthy controls	*p*-value
*N* (%)	38 (66%)	20 (34%)	
Right body side			
CDT (cold detection threshold) [°C]	29 (25–30)	30 (28–31)	0.021
WDT (warm detection threshold) [°C]	37 (35–39)	36 (35–37)	0.056
CPT (cold pain threshold) [°C]	11 (3–24)	21 (9–26)	0.16
HPT (heat pain threshold) [°C]	47 (42–50)	46 (41–48)	0.15
MDT (mechanical detection threshold) [mN]	7 (4–17)	6 (3–13)	0.31
MPT (mechanical pain threshold) [mN]	30 (10–62)	15 (9–40)	0.16
MPS (mechanical pain sensitivity)	9 (5–19)	5 (1–8)	0.001
DMA (dynamic mechanical allodynia)	0.0 (0.0–0.4)	0.0 (0.0–0.0)	0.006
WUR (wind-up ratio)	0.3 (0.2–0.5)	0.4 (0.2–0.6)	0.59
PPT (pressure pain threshold) [kg/cm^2^]	6 (5–8)	8 (6–10)	0.007
Left body side			
CDT (cold detection threshold) [°C]	29 (27–30)	30 (29–31)	0.002
WDT (warm detection threshold) [°C]	37 (35–38)	36 (35–38)	0.09
CPT (cold pain threshold) [°C]	19 (5–26)	24 (18–27)	0.12
HPT (heat pain threshold) [°C]	45 (41–49)	43 (40–47)	0.045
MDT (mechanical detection threshold) [mN]	7 (3–18)	6 (3–14)	0.66
MPT (mechanical pain threshold) [mN]	23 (11–42)	15 (9–29)	0.23
MPS (mechanical pain sensitivity)	10 (4–18)	4 (1–7)	0.002
DMA (dynamic mechanical allodynia)	0.0 (0.0–0.7)	0.0 (0.0–0.0)	0.003
WUR (wind-up ratio)	0.4 (0.3–0.6)	0.3 (0.2–0.5)	0.19
PPT (pressure pain threshold) [kg/cm^2^]	6 (5–8)	7 (6–9)	0.047

Otherwise indicated, values are expressed as median and IQR. Mann–Whitney-*U*-test for comparisons was used.

**Table 5:** Comparison of quantitative sensory testing in patients with cILD without thoracic pain and healthy controls.

Group	ILD patients without thoracic pain	Healthy controls	*p*-value
*N* (%)	40 (67%)	20 (33%)	
Right body side			
CDT (cold detection threshold) [°C]	29 (27–30)	30 (28–31)	0.07
WDT (warm detection threshold) [°C]	36 (35–38)	36 (35–37)	0.41
CPT (cold pain threshold) [°C]	2 (0.0–25)	21 (9–26)	0.052
HPT (heat pain threshold) [°C]	48 (44–50)	46 (41–48)	0.025
MDT (mechanical detection threshold) [mN]	5 (2–10)	6 (3–13)	0.66
MPT (mechanical pain threshold) [mN]	34 (20–95)	15 (9–40)	0.001
MPS (mechanical pain sensitivity)	2 (0.7–7)	5 (1–8)	0.058
DMA (dynamic mechanical allodynia)	0.0 (0.0–0.0)	0.0 (0.0–0.0)	0.054
WUR (wind-up ratio)	0.2 (0.1–0.5)	0.4 (0.2–0.6)	0.051
PPT (pressure pain threshold) [kg/cm^2^]	7 (6–9)	8 (6–10)	0.10
Left body side			
CDT (cold detection threshold) [°C]	30 (27–30)	30 (29–31)	0.005
WDT (warm detection threshold) [°C]	36 (34–38)	36 (35–38)	0.93
CPT (cold pain threshold) [°C]	5 (0.0–26)	24 (18–27)	0.009
HPT (heat pain threshold) [°C]	47 (43–49)	43 (40–47)	0.005
MDT (mechanical detection threshold) [mN]	6 (3–15)	6 (3–14)	0.79
MPT (mechanical pain threshold) [mN]	50 (22–116)	15 (9–29)	<0.001
MPS (mechanical pain sensitivity)	2 (0.4–5)	4 (1–7)	0.017
DMA (dynamic mechanical allodynia)	0.0 (0.0–0.0)	0.0 (0.0–0.0)	0.34
WUR (wind-up ratio)	0.3 (0.2–0.6)	0.3 (0.2–0.5)	0.68
PPT (pressure pain threshold) [kg/cm^2^]	7 (5–8)	7 (6–9)	0.17

Otherwise indicated, values are expressed as median and IQR. Mann–Whitney-*U*-test for comparisons was used.

## 4. Discussion

This is the first study specifically investigating thoracic pain in patients with chronic interstitial lung disease. Thoracic pain occurred in 48.7% of patients with chronic interstitial lung disease, most frequently in those with pulmonary sarcoidosis. Moreover, we found an association of thoracic pain with lung function and quality of life. Pain intensity did not differ between patients with pulmonary sarcoidosis and the other patients with chronic interstitial lung disease.

Thoracic pain seems to be an underestimated symptom in patients with chronic interstitial lung disease and, in general, knowledge on thoracic pain in patients with interstitial lung disease is scarce. A recent study reported that the prevalence of pain in ILD patients was 62% compared to 25% in healthy controls, with thoracic pain being the most frequent form (46%), followed by joint and limb pain (27). In that study, the occurrence of chest pain was higher in patients with idiopathic pulmonary fibrosis than those with CTD-ILD. Moreover, an association was found between intensity of pain, dyspnea, and quality of life.

In our study, pain usually occurred spontaneously, and appeared to be related to the disease itself rather than to previous interventions. It is likely that this kind of pain is associated with the fibrotic involvement and consequent thickening of the pleura, especially in idiopathic pulmonary fibrosis, where the fibrotic changes are predominantly localized in the subpleural area. In patients with pulmonary sarcoidosis, on the other side, thoracic pain may be related to an involvement of small nerve fibers. It has been reported that small fiber neuropathy occurs in 30%–50% of sarcoidosis patients, with pain and paresthesia being the most common symptoms (28, 29). Further studies are needed to elucidate the origin of thoracic pain in ILD patients.

With regard to pain characterization, significant signals in specific tests among the whole quantitative sensory testing were observed in cILD patients. Comparing cILD patients with thoracic pain and healthy controls, the observed significant differences in cold detection threshold, heat pain threshold, dynamic mechanical allodynia and pressure pain threshold mean a lower sensitivity to thermal testing and increased sensibility to pressure at the thenar. In addition, the lower values in cold detection threshold and cold pain threshold, and higher values in heat pain threshold in patients without thoracic pain compared to healthy controls underline the lower sensitivity to thermal stimuli of cILD patients. In contrast, patients without thoracic pain point out higher values in mechanical pain threshold as well as lower values in numeric rating scale in mechanical pain sensitivity compared to healthy controls, which discloses a lower sensitivity against pinprick stimulation and contrasts with the higher sensitivity to pinpricks in patients with thoracic pain. In summary, thoracic pain in ILD compared to healthy controls seems to be characterized by decreased perception and pain sensitivity in response to thermal stimuli. Moreover, patients with thoracic pain compared to healthy controls showed an increased sensitivity to pinprick stimuli and pressure pain.

We observed that subjects under long-term corticosteroid treatment had a lower sensitivity to temperature and pressure during quantitative sensory testing. This may be explained by the analgesic effect of corticosteroids as described in previous studies (30).

Similarly to the study by Shen et al. (27), we did not find a correlation between lung function tests and the intensity of thoracic pain. Nevertheless, positive correlations between total lung capacity and sensitivity to stimuli, i.e., cold detection threshold as well as pressure pain threshold in all cILD patients (not only those with thoracic pain) were found. The positive correlation between total lung capacity and cold detection threshold shows that patients with mild or no ventilatory restriction can perceive coldness similarly to healthy subjects, whereas a more pronounced restriction in patients with advanced ILD seems to be associated with a pathologically reduced perception of coldness. This may indicate that the fibrotic changes of the lung tissue affect nerval pathways of the thorax.

The positive correlation between total lung capacity and pressure pain threshold indicates that higher pressure on the thenar is necessary to trigger a painful sensation in patients with better values in total lung capacity. This means that patients with more pronounced restriction have a hypersensitivity to pressure at the thenar. In patients with sarcoidosis, the hypersensitivity at the thenar could possibly be explained by a generalized sensitivity to pain in other parts of the body associated with a chronic pain syndrome or small fiber neuropathy (31).

Thoracic pain seems to cause a limitation of mental well-being in patients with thoracic pain. An association between pain in patients with ILD and impaired quality of life was observed by Shen et al. (27). Our study confirms recent investigations in patients with pulmonary sarcoidosis, in whom the loss of mental well-being and concentration impairment have been reported with high frequency (32). Similar to the observations of Shen et al. (27), by using the painDETECT questionnaire we could detect more pain areas in patients with thoracic pain than in patients without thoracic pain. This was reflected by the significantly higher consumption of non-opioid analgesics in patients with thoracic compared to those without. Since medication burden is an important factor impacting patients quality of life, early pain identification and management could lead to a better preservation of quality of life in ILD patients.

This study has several limitations. First, the sample size of the present study does not allow to perform subgroup analyses or prediction model to investigate risk factors for thoracic pain. Second, we did not include patients with lung diseases other than ILDs as a control group. This may lead to overestimation of thoracic pain as symptom in ILD, as the prevalence of pain in patients with chronic obstructive pulmonary disease is reported with 32%–60% (33). Moreover, it is known that the prevalence of fibromyalgia is higher in patients with sarcoidosis. Unfortunately, fibromyalgia was not systematically investigated in our cohort. Furthermore, the lack of a matched control group may lead to biased results since the controls are younger and more likely to be female. Third, the cross-sectional study design does not allow drawing any conclusion about long-term consequences of thoracic pain on lung function, quality of life, or disease course. Moreover, we were not able to analyze the temporal relation with onset of ILD symptoms since data on duration of thoracic pain were not available. Fourth, previous treatment with corticosteroids and analgesics could have led to underestimation of thoracic pain in our patients’ population. Finally, in quantitative sensory testing it is difficult to distinguish between faked and true loss or gain of sensation as well as central and peripheral abnormalities can lead to the same deficit in measurement.

This study was performed to investigate prevalence and risk factors of thoracic pain in patients with chronic interstitial lung disease. Thoracic pain mostly occurs spontaneous as a frequent symptom in chronic interstitial lung disease patients, especially in pulmonary sarcoidosis. Timely identification of thoracic pain may allow starting symptomatic treatment at early stage, before impairment in quality of life occurs.

## Data availability statement

The raw data supporting the conclusions of this article will be made available by the authors, without undue reservation.

## Ethics statement

The studies involving human participants were reviewed and approved by Ethics Committee of the Medical Faculty of the University Duisburg-Essen. The patients/participants provided their written informed consent to participate in this study.

## Author contributions

MS and FB are responsible for all content of the manuscript, contributed substantially to the study design, data analysis and interpretation, and the writing of the manuscript. SK contributed substantially to the study design and the writing of the manuscript. JF-B contributed substantially to the data analysis and the writing of the manuscript. GW contributed substantially to the study design, data analysis, and the writing of the manuscript. UC contributed substantially to the data interpretation and the writing of the manuscript. All authors contributed to the article and approved the submitted version.

## Funding

This study received funding from Boehringer-Ingelheim Pharma GmbH & Co. KG. The funder was not involved in the study design, collection, analysis, interpretation of data, and the writing of this article or the decision to submit it for publication.

## Conflict of interest

FB and UC received speaker and advisor honoraria, travel and research grants from Boehringer Ingelheim Pharma, not related to this study.

The remaining authors declare that the research was conducted in the absence of any commercial or financial relationships that could be construed as a potential conflict of interest

The handling editor PS declared a past co-authorship with the author FB.

## Publisher’s note

All claims expressed in this article are solely those of the authors and do not necessarily represent those of their affiliated organizations, or those of the publisher, the editors and the reviewers. Any product that may be evaluated in this article, or claim that may be made by its manufacturer, is not guaranteed or endorsed by the publisher.

## Figures and Tables

**Table:** 

CDT	cold detection threshold
COPD	chronic obstructive pulmonary disease
CPT	cold pain threshold
cILD	chronic interstitial lung disease
DIP	desquamative interstitial pneumonia
DLCO	diffusing capacity for carbon monoxide
DMA	dynamic mechanical allodynia
EAA	exogenous allergic alveolitis
FEV_1_	forced expiratory volume in 1 s
HCs	healthy controls
HP	hypersensitivity pneumonitis
HPT	heat pain threshold
HRCT	high resolution computed tomography
IIP	idiopathic interstitial pneumonias
ILD	interstitial lung disease
IPF	idiopathic pulmonary fibrosis
IVC	inspiratory vital capacity
MDT	mechanical detection threshold
MPS	mechanical pain sensitivity
MPT	mechanical pain threshold
NRS	numerical rating scale
NSIP	nonspecific interstitial pneumonia
paO_2_	oxygen partial pressure
PAP	pulmonary alveolar proteinosis
PPT	pressure pain threshold
QoL	quality of life
QST	quantitative sensory testing
RBILD	respriratory bronchiolitis
TLC	total lung capacity
TP	thoracic pain
TP+	patients with thoracic pain
TP−	patients without thoracic pain
WDT	warm detection threshold
WUR	wind-up ratio
