# Supplementary figures and images for: Thoracic pain in patients with chronic interstitial lung disease—an underestimated symptom
Source: Front Med (Lausanne). 2023 May 5;10:1147555. doi: 10.3389/fmed.2023.1147555 (PMC10196162; doi:10.3389/fmed.2023.1147555)

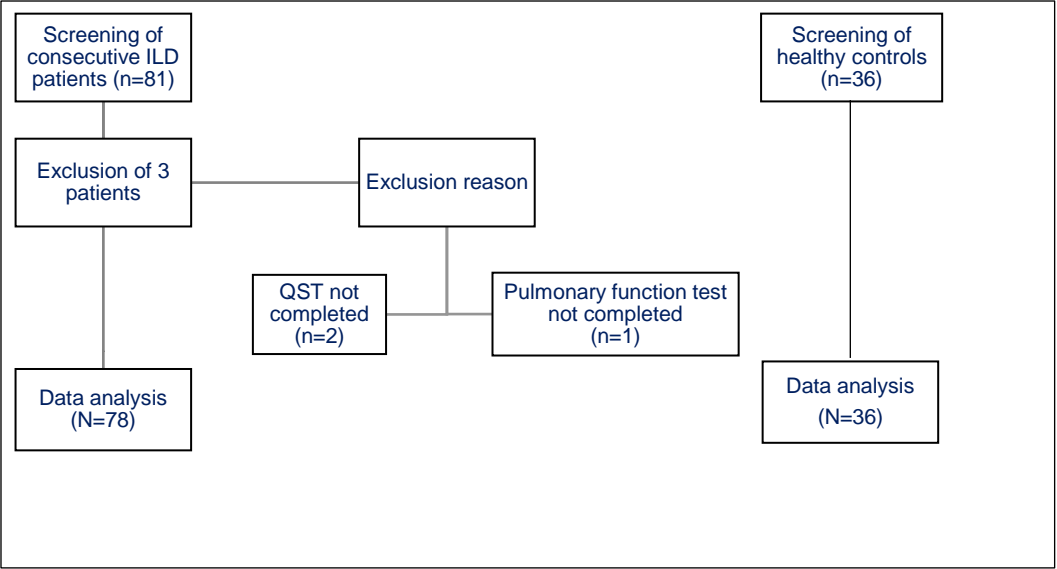

Supplement: Supplementary file 1 [file Data_Sheet_1.PDF]
